# Report of the Highlands and Islands Medical Service Board

**Published:** 1917-06-16

**Authors:** 


### The Hospital

The Workers' Newspaper of Administrative Medicine and Institutional Life, Administration, National Insurance and Health.

No. 1619, Vol. LXII. SATURDAY, JUNE 16, 1917. PRICE ONE PENNY

### REPORT OF THE HIGHLANDS AND ISLANDS MEDICAL SERVICE BOARD.

The report of this Board is always interesting, and the work it recites must command the good will of all who recognise the special difficulties of medical practice in the more remote parts of the Northern Kingdom. The long distances to be accomplished, the absence of means of communication, and the lowly circumstances of a large proportion of the population stand in the forefront of the problem, which is concerned alike with an adequate medical service for the people and something like reasonable remuneration for the practitioner. Perhaps it may be fairly said that these are merely opposite sides of one and the same shield. Such an admission would not involve any reflection on the medical profession, for here, as elsewhere, the labourer is worthy of his hire. Thus even for men whose inclinations lead them to the wide spaces of the Scottish Highlands, it is necessary to establish a living wage, for failing this the quantity or quality of the supply must inevitably diminish. What is true of doctors is true also of nurses; economic considerations exercise their just influence, and if the tune is to be called there must be a guarantee that the piper will be paid. Such was the centre of the position when the Board, which now issues its third report, was constituted in 1913. The medical and nursing services for the districts coming under the care of the Board were notoriously defective; hospital facilities and opportunities for consultations hardly existed; and as a result there occurred without question unnecessary suffering and disaster to individual sufferers. The commencement of the work was not wanting in happy omens. Parliament provided a sum of money; the Board sketched out a programme which was based upon personal experience of the actual conditions; local authorities were stimulated in the direction of duty; and, subject to some exceptions, the medical profession received an undertaking which made it possible for them to give reasonable attendance on poorer patients even when these were resident in the more remote districts. Still greater things were anticipated—such, for example, as a freer service of nurses, the establishment of small hospitals, and the co-operation of practitioners engaged in special branches of practice. That all these schemes involved much work and many journeyings is obvious, but fortunately the Board possessed some members who combined patriotic enthusiasm with a keen sense of practical affairs, and under these influences the future seemed bright with promise. If the work did not touch the world of fashion or even that of controversial politics, it certainly came near to a body of citizens who are not the least estimable members of our democratic State.

Now the Board is able to tell us how it has fared in its third year of service. And the tale is in some measure a qualified one. Here, as elsewhere, the war has exercised its limiting and restricting influence. If in existing conditions there is a short supply of doctors for the civil needs of our great industrial centres, it is hardly surprising to find that a similar limitation applies to the Highlands where, relative to the areas to be covered, there is never a surplus. Then, as all will allow, the fiery cross of war makes a special appeal to the Highlander, and the present emergency has been no exception to the rule. From glen and moor he has gone in his thousands, and, as many a stricken field proclaims, he has gone to some purpose. Among others the doctors have gone, part as holding commissions in the Territorial Army, and part in response to the calls of the War Office for assistance and co-operation. Substitutes are hard to find for any kind of medical work just now, and they have been sadly to seek for practices in the Highlands and Islands. The Board has had but a limited supply of cloth, and naturally therefore in certain directions the garment has been of scanty proportions. In some places where formerly a resident doctor has been one of the resources of civilisation a less continuous arrangement has alone been possible, and an occasional medical visitor has had to do his best to meet the local demands. With the *personnel* for the nursing schemes the Board have been more fortunate in spite of the multiplied demands from the military authorities, and they are able to report the establishment of five new nursing associations and the extension of two which had been founded in previous years. These activities mean a definite addition to the number of nurses employed, and all interested in the Highlands will learn of this development with much satisfaction. In this direction, as well as in regard to the guarantee of fees to medical practitioners, the Board has wisely insisted on the practical co-operation of local bodies. Help is given not to relieve these bodies from the calls of responsibility and duty, but to arouse them to opportunities. In a word, what is offered by the Board is conditioned by what is undertaken locally, and in this way the Board has a double efficacy. It not only brings enterprise itself, but it is a cause of enterprise in others. Just now it is compelled perforce to pitch its operations on a lower level than its earlier ambitions suggested. But, in the language of the tragic stage—a time will come. The Highlanders and their doctors—not all of them, alas!—will come back to their glens. With peace, thoughts about nursing schemes, and midwives, and expectant mothers, and the welfare of children will again take their natural course, and all the more freely for the present hindrances. The manhood of the race has gone down into the battle, and the new generation will have not only its own burden to bear but many gaps to fill. Good doctoring and good nursing are helps to this end, and the Board which fosters them merits a hearty God-speed.

